# The Correlation between Thermal and Noxious Gas Environments, Pig Productivity and Behavioral Responses of Growing Pigs

**DOI:** 10.3390/ijerph8093514

**Published:** 2011-08-25

**Authors:** Hong Lim Choi, Sang Hwa Han, Louis D. Albright, Won Kyung Chang

**Affiliations:** 1 Department of Agricultural Biotechnology, Seoul National University, Seoul, 151-921, Korea; E-Mail: sanghwa1002@hotmail.com; 2 Department of Biological and Environmental Engineering, Cornell University, New York, NY 14853, USA; E-Mail: lda1@cornell.edu; 3 Department of Animal Biotechnology, National Livestock Research Institute, Suwon, 441-706, South Korea; E-Mail: changwk@rda.go.kr

**Keywords:** growing pigs, thermal environment, noxious gas, average daily gain, feed efficiency, behavioral responses

## Abstract

Correlations between environmental parameters (thermal range and noxious gas levels) and the status (productivity, physiological, and behavioral) of growing pigs were examined for the benefit of pig welfare and precision farming. The livestock experiment was conducted at a Seoul National University station in South Korea. Many variations were applied and the physiological and behavioral responses of the growing pigs were closely observed. Thermal and gas environment parameters were different during the summer and winter seasons, and the environments in the treatments were controlled in different manners. In the end, this study finds that factors such as Average Daily Gain (ADG), Adrenocorticotropic Hormone (ACTH), stress, posture, and eating habits were all affected by the controlled environmental parameters and that appropriate control of the foregoing could contribute to the improvement of precision farming and pig welfare.

## 1. Introduction

Environmental parameters in livestock houses, such as temperature and humidity, have been typically used as indicators to evaluate the indoor aerial environment for the different growth phases of livestock, based on their productivity. Thermal environmental parameters, however, may not be accurate indicators because they do not directly reflect the physiological responses of the animals to their surroundings. From the animal’s point of view, a difference may exist between actual physiological comfort and what is considered an optimum range of physical environment parameters.

Ambient temperature is a most important environmental parameter for pigs because it directly affects their productivity. Temperatures beyond the acceptable range reduce pig growth rate by decreasing feed intake. Such an effect negatively affects the growth period of the pig and reduces its productivity level. Nienaber *et al.* reported no significant differences in performance for temperatures ranging from 5 °C to 15 °C, and average daily gain (ADG) was not significantly affected when temperatures ranged between 5 °C and 20 °C. Although ADG was not significantly correlated to ambient temperature, and ADG variations were not significant at different ambient temperatures, the optimum temperature of 20 °C provided the highest ADG for pigs [1]. Lemay also studied the ADG of pigs in two temperature-set point patterns: 25.9–19.7 °C for control and 25.7–18.1 °C for treatment in a pilot experiment over the summer months [2]. ADG in the treatment increased by 5.2% compared to the control. However, no significant statistical difference of ADG was found (P > 0.05) between the two trials.

Evaporative losses at optimum temperatures were less than 10% of total heat loss. Evaporation becomes an important factor of heat loss when the air temperature approaches the pig surface temperature and sensible losses are reduced [3]. As such, humidity levels are important at high temperatures. Rafai and Papp found that relative humidity only affected the heat production or heat balance of pigs when temperatures were above 33 °C and humidity levels were over 90% [3]. At 29 °C (a high temperature), fully grown up pigs showed no difference in growth characteristics at humidity levels of 35% or 85% [4]. Thus, humidity has little or no direct role in the performance of growing pigs when the air temperature is below 30 °C. Nevertheless, higher levels of humidity at higher temperatures may not be acceptable to farm personnel [5], the building structure [6], and the spread of diseases [7].

Noxious gases such as ammonia (NH_3_) and carbon dioxide (CO_2_), can affect an animal’s health and productivity in several direct and indirect ways. The duration of exposure, the concentration levels and the simultaneous presence of other aerial pollutants or environmental factors can cause significant harm. While acute exposure for only several minutes in a day may cause harm, chronic exposure is considered being consistently exposed for several days or months. An animal’s behavior during acute or chronic exposure to ammonia results in a number of physiological mechanisms and reflects how the animal ‘feels’ about being exposed to the noxious gas. Morrison observed a growing pig facility by adjusting ammonia ranges from 17.7–50.0 ppm and carbon dioxide ranges 4,950–10,000 ppm while keeping a ventilation rate of 2–4 ACH (air change per hour) [8]. Such ammonia and carbon dioxide concentrations far exceeded the recommendations by Donham [9] who suggested, from his study on association of environmental air contaminants with finishers’ disease and productivity, that concentrations up to 7 ppm NH_3_ and 1,540 ppm CO_2_ were not harmful. Both researchers concluded that ammonia concentrations in commercial facilities were unlikely to be significant and stated that the level of ammonia present in commercial buildings appears to be of no great consequence to the animal’s perceived well-being. Another researcher, Stombaugh [10], investigated the productivity of pigs at four ammonia concentration levels (10, 50, 100, 150 ppm). He found that feed consumption of growing pigs and ADG showed significant adverse effects, but feed conversion was not affected at the higher ammonia levels (100, 150 ppm).

Massabie reported that high levels of gases (NH_3_, CO_2_) could decrease appetite and ADG at temperatures of 17 °C, 20 °C, 24 °C or 28 °C. The experiment was performed on a total of 672 growing and finishing pigs [11]. Massabie carried out two experiments, each involving 192 feeders, to determine the effect of air movement and ambient temperature on the performance and behavior of pigs [12]. The treatments included three ambient temperatures (28 °C, 24 °C and 20 °C) combined with two air velocities (still air or 0.56 m/s increasing up to 1.3 m/s). Relative humidity remained constant at 65–70%. Fresh air renewal rates and hygrometry were the same for all treatments. Animal performance (weight gain, feed conversion) was measured throughout the entire trial period. At temperatures of 28 °C and 24 °C, increasing air velocity improved growth rate and feed consumption but decreased feed efficiency. At an ambient temperature of 20 °C, high air velocity increased food consumption but both growth rate and feed efficiency decreased. Pigs housed at 24 °C with still air were less active and the lying area was greater than such pigs housed at a cooler environmental temperature (20 °C) or with higher air movement. At an ambient temperature of 20 °C with high air velocity, 90% of the pigs began to huddle together. Interactive assessment and control of swine thermal comfort was observed and developed by using real-time computer image analysis of pig resting patterns [13]. The researchers were able to develop an image processing algorithm system by using paper-cut pigs in laboratory settings and briefly validating using live pigs in production settings.

The purpose of this study was to examine the correlation between thermal and gas environment parameters, and the productivity, physiological, and behavioral responses of growing pigs for well-being and precision farming.

## 2. Materials and Methods

### 2.1. Experimental Bays and Animals

This study was performed at the collegiate livestock experiment station at Seoul National University, South Korea. The summer experiment was conducted for a month that started on 1 July 2003, and ended on 31 July 2003. The winter experiment began on 10 February 2004 and lasted through 3 March 2004. The bay building structure for the experiments was 4.5 m wide and 8.5 m long, and divided into two rows of pens with a central alley 0.9 m wide. Each row comprised five pens and each pen (1.7 m in width, 1.8 m in length, 0.6 m in height, and total unit area of 0.765 m^2^) housed four growing pigs. The bay for Treatment 1 was 5.6 m wide and 8.5 m long, and divided into two rows of pens with a central alley 0.8 m wide. Each row was comprised of five pens and each housed four growing pigs in a space of 1.7 m in width, 2.4 m in length, and 0.6 m in height. Its stocking density was 1.02 m^2^/head, which was one-third more space than in the Treatment 2 pens.

**Figure 1:**
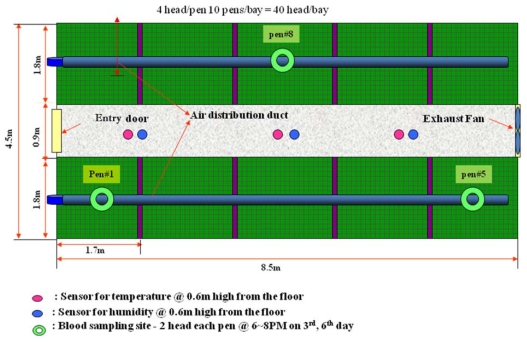
The sampling locations of temperature, humidity, gases, and blood in the experiment bay (Treatment 1).

**Figure 2:**
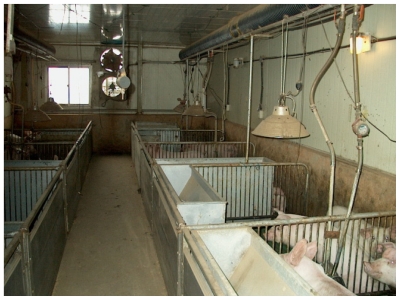
The experimental bay (Treatment 1) for the growing pigs in winter.

The growing pigs for the experiment were three-way crossbred: Landrace × Yorkshire × Duroc. The confinement house comprised ten pens, each of which housed four pigs about 60 days old, with average weights of 60 to 65 kg. The weight of the pigs was averaged for the twelve pigs in the three pens shown in Figures 1 and 2. The same was done for blood sampling in both bays, each of which housed 40 growing pigs. They were fed diets *ad libitum*, and were allowed to drink freely from nipple waterers attached on the fence. Wastes were collected through a plastic flooring system into a pit and the slurry was drained when the pigs were moved to the next growth phase house.

### 2.2. Animal Behavior Observation

The pigs were observed for their behavioral responses to different microclimates (Treatment 1 and Treatment 2 during the summer and winter). The number of pigs in each bay was counted and their pose (lying, standing, sitting, drinking, eating, and fighting) was recorded at every hour from (10:30 AM through 6:30 PM) on the 3rd and 6th day of each week. Only a few minutes were required to count the pigs posing different behaviors in the bays.

### 2.3. Sample Parameters, Their Measurements and Instruments

Figure 1 shows the sampling locations of the experiment bay for temperature, humidity and blood samples. Thermal environment parameters—temperature and humidity—were automatically recorded at 0.6 m above the floor. The measurements were stored every hour by the hybrid recorder (NEC 3500, 64 channels) for the entire period of the experiment.

The concept of the enthalpy of moist air is included in this study through using the air enthalpy index (AEI), developed by Makoto [14]. The AEI is determined by multiplying the air temperature by its relative humidity. The AEI comfort level of growing pigs is recommended to be within the range of 1,000 to 1,500. The AEI method was included in this study and correlated to other environmental parameters.

Air speed was periodically measured by a Kanomax (Andover, NJ, USA) Model No. 6112 anemometer (range 0~50 m/s). CO_2_, and NH_3_ were measured with a Gastec (Kanegawa, Japan) GV-100S precision sampling pump (range 0~30 mg/L) placed at the locations shown in Figure 1. Mesurments were recorded every three hours from 10 AM to 6 PM on the day of the experiment. Blood samples were taken once at 6 PM from two pigs each in the No.1, No. 5 and No. 8 pens, shown in Figure 1, in both the Treatment 1 and Treatment 2 bays. Average Daily Gains, ADG, (ADG = (Weight_t+Δt_ – Weight_t_)/Δt) and Feed Efficiencies, FE, (FE = Weight Gain_kg/d_/Feed Intake_kg/d_), were calculated to evaluate productivity.

### 2.4. Modification of Aerial Environment for the Treatments

#### 2.4.1. Summer

The summer aerial environment was purposely modified by turning fans off and providing no ventilation in the treatment bay when outside air temperature varied between the range of 20.5–27.7 °C with a mean relative humidity of 75% in July, 2003. No ventilation in Treatment 2 naturally led to higher indoor humidity, noxious odor concentration, frequent abnormal behaviors and greater stress symptoms. The experiment bay for Treatment 1 maintained a ventilation rate of 1.68 m^3^/s as normal, which is 95% of the fan capacity.

#### 2.4.2. Winter

The winter aerial environment in Treatment 2 was purposely modified by having fans operate at a doubled ventilation rate of 0.530 m^3^/s (Treatment 1 maintained a minimum ventilation rate of 0.265 m^3^/s which is equivalent to 15% of the fan capacity). In other words, excessive ventilation was applied to the Treatment 2 bay when outside air temperature varied within the range of −3.0~7.5 °C, with a mean relative humidity of 56%, in February 2004. This resulted in a chilly environment for the growing pigs but it lowered indoor humidity, noxious odor concentration, frequent abnormal behavior, and higher stress symptoms.

### 2.5. ACTH Measurement by Blood Sample

The blood samples (approximately 15 mL) were collected using heparin syringes in Treatment 1 and Treatment 2 bays at 6:00 PM in both seasons. Blood tubes were stored in ice cubes and subsequently centrifuged with the resultant plasma divided into aliquots and stored at −20 °C and transferred to the special research institute for the assay of ACTH, which is produced in response to biological stress.

### 2.6. Statistical Analysis

The t-test utilizing SPSS (version 17.0) was performed to assess whether the means of two groups were statistically different. This analysis is appropriate to compare the means of two groups, and especially appropriate as the analysis for the post test-only two-groups randomized experimental design. Duncan’s multiple range tests were also performed to measure the significance among parameters in the Pearson’s correlation analysis.

## 3. Results and Discussion

### 3.1. Summer

The mean values, which include air speed, temperature, and humidity for thermal environment; NH_3_ and CO_2_ for indoor noxious gas environment; ACTH for animal welfare; and ADG and FE for production performance, are shown in Table 1. In addition, animal behaviors such as lying (lateral and sternal posture), standing, sitting, drinking, eating, and fighting, were observed to relate behavioral response to different environments. The t-test for the two independent groups, Treatment 1 and Treatment 2, were used to assess whether the means and variance of the two groups differed statistically. Treatment 2, with higher density of 0.62 m^2^/head, was manipulated (by not operating the ventilation system) to maintain a harsh thermal environment, whereas ventilation for Treatment 1, with lower density of 0.75 m^2^/head, was maintained properly to investigate how thermal and gaseous environments affect productivity, animal physiology, and animal behavior. Temperature positively correlated to AEI in both Treatments. This is a natural result for AEI, which is calculated by multiplying temperature and humidity. The results of the t-test analysis are shown in Table 1. The significance levels of all parameters of the two groups were more than 0.01 except ‘fighting’—which was not significant.

**Figure 3:**
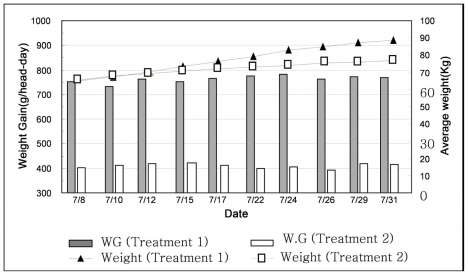
Weight variations and weight gains for Treatments 1 and 2 during summer.

The weight changes in both bays can be formulated mathematically as follows:

(1) $${\text{W}}_{\text{t}}=\alpha \;\text{t}+\beta$$

where W_t_ is weight at time t in kg, α and β are empirical parameters, and t is production time in days. The regression equation was W_t_ = 0.776 t + 60.99 (R^2^ = 0.993) for weight change in the Treatment 1 bay, and W_t_ = 0.463 t + 67.18 (R^2^ = 0.963) for the Treatment 2 bay, shown in Figure 3. The derivative of pig weight with respect to time in Treatment 1 was calculated to be 0.776 kg/day, but only 0.436 in Treatment 2, which was 45% lower. Since the rate of ADG differs with pig growth stage, it would be very interesting to investigate further the relation of the growth stage and ADG.

In Treatment 1, higher CO_2_ concentration correlated positively to ADG (P < 0.05) and FE (P < 0.01) in Table 2. This is not in accordance with the results of previous research work reviewed in this study [11]. This may be attributed to the limited number and range of data used by such studies. As a result, the statistical software could have mechanically produced unrealistic outcomes. FE was positively correlated with ADG at a significant level of 95%. This can be simply explained by the intrinsic definition of both parameters. However FE is also positively correlated with temperature and CO_2_ concentration. Such incompatibility is assumed to be caused by data limitation as mentioned above. No statistically significant correlations were found in Gas Environment (NH_3_, CO_2_) and productivity parameter (ADG, FE) in Treatment 2 in Table 3.

**Figure 4:**
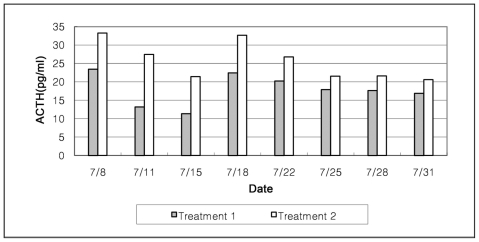
ACTH variations for Treatments 1 and 2 during summer.

ACTH was observed to relate thermal and gas environments directly to pig productivity. ACTH is known to be a reliable indicator of animals stress. Higher ACTH concentrations may be interpreted as animals having higher stress levels. The mean ACTH was 26.27 pg/mL in Treatment 2, which is 64.5% higher than Treatment 1 of 15.97 pg/mL, as shown in Figure 4.

Regarding behavioral response to thermal and gas environments in the treatments, significantly more pigs were lying (P < 0.05), and not sitting (P < 0.05) at moderate temperature ranges in Treatment 1. This is believed to be caused by higher ADG and FE. The higher productivity of pigs (fattening their body weight) was significantly well represented when displaying the lying pose.

In hot, humid, NH_3_-rich environment in the Treatment 2, more pigs were lying (P < 0.01), and not sitting (P < 0.01) nor fighting (P < 0.05). This may be due to severe exhaustion or the harsh environment setting. The comparatively higher ADG, made pigs stand (P < 0.05) and they only drank frequently (P < 0.05) but did not eat as much (P < 0.05), ultimately leading to decreased levels of ADG.

### 3.2. Winter

Comparisons of Treatment 1 to Treatment 2 during the winter are shown in Table 4. In the thermal environment, the mean air speed, temperature, and humidity, were 0.89 m/s, 10.7 C and 51.9% in Treatment 2, while the parameters were 0.18 m/s, 19.5 C, and 75.4%, respectively, in Treatment 1. Providing more air exchanges (doubling the minimum ventilation rate) in the Treatment 2 bay lowered the indoor temperature. However, it also diluted noxious gases and water vapors to a greater extent and so lowered NH_3_ and CO_2_ concentrations, and humidity levels. Treatment 2 is a trade-off situation, environmentally. Although the air speed became higher and the indoor temperature was lower in Treatment 2, all the environment parameters still fell within acceptable ranges.

The results of the t-test analysis are shown in Table 4. The significance levels of the thermal and gas environments, and productivity parameters of the two independent groups were at the 99.9% level. The behavioral parameters were not as they did not show much significance. The means and variances of Treatment 1 and Treatment 2 were proven to be statistically not the same, except behavioral parameters, unlike the summer case.

**Figure 5:**
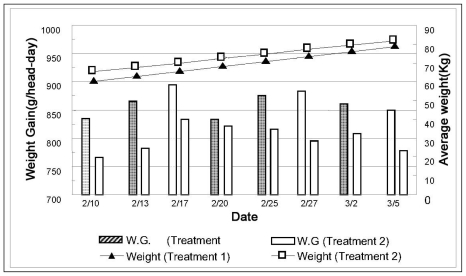
Weight variations and weight gain for Treatments 1 and 2 during summer.

Humidity positively correlated to AEI in both treatments. As mentioned before, it seems very natural if the definition of AEI is carefully examined because it is determined by multiplying temperature and relative humidity. The linear equation, W_t_ = 0.776t + 60.09 (R^2^ = 0.994) was formulated to predict the weight change of growing pig in the Treatment 1 bay, and W_t_ = 0.702t + 65.18 (R^2^ = 0.994) in Treatment 2 bay, shown in Figure 5. The slope in Treatment 2 is 9.5% lower than that in Treatment 1. A higher slope indicates higher ADG of pig weight during the winter growing phase. Although the temperature difference between Treatment 1 and Treatment 2 was about +6 °C in summer and −9 °C during the winter, their productivity in winter decreased by only 9.5%, compared to that during the summer, which decreased by 45%. This may be attributed to improvement of the gas environment during the winter, and reduced humidity to 52% (excessive ventilation rate was applied).

A statistical analysis was also performed for the winter group to examine how the thermal and aerial environments affected productivity, physiology, and behavior. The correlation coefficient, R and probability (P-values) for Treatment 1 are displayed in Table 5, and Treatment 2 in Table 6.

**Figure 6:**
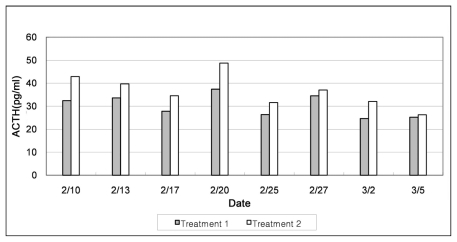
ACTH variations for Treatments 1 and 2 during winter.

The mean ACTH in Treatment 2 was 36.61 pg/mL, which was 21% higher than for Treatment 1 (30.24 pg/mL as shown in Figure 6). This result is consistent with the outcomes of ADG, which is one of the direct productivity parameters in this study.

In Treatment 1, correlations of gas environment parameters were not significant with productivity. However, in Treatment 2, humidity positively correlated with CO_2_ concentration, and temperature positively influenced to ADG (P < 0.05) which fluctuated 10.7 ± 1.1 °C. In such temperature ranges, warmer temperature raises ADG.

The mean ACTH in the treatment was 36.61 pg/mL, which was 21% higher than Treatment 1 (30.24 pg/mL as shown in Figure 6). This result is consistent with the outcomes of ADG, which again is one of the direct productivity parameters in this study. With regard to the behavioral response to thermal and gas environments in both treatments, in Treatment 1, significantly more pigs were eating (P < 0.01) at moderate temperature ranges. More pigs were lying, than standing, which seemed normal in Treatment 1. In Treatment 2, at a lower temperature, fewer were drinking (P < 0.05), and fighting (P < 0.05). Higher stress levels lend pigs to take a more sternal recumbency posture (P < 0.05) and fewer in standing (P < 0.05). Among behavioral responses, more pigs tended to fight for access to the only drinking nipple in the pen.

## 4. Conclusions

This study was performed to investigate the correlation between thermal and noxious gas environment parameters, and productivity, physiological, and behavioral responses of growing pigs. The environments were modified by controlling ventilation rates during the summer and winter. Results may be summarized as follows:

*Thermal and Gas Environments-ADG*: During summer, the ADG rate was 67% higher in Treatment 1 than in Treatment 2. During winter, the ADG was c. 10% higher in Treatment 1 than in Treatment 2. This indicates that hot and humid environments with higher noxious gas concentrations or colder thermal environments depress pig productivity.
*Thermal and Gas Environments-ACTH*: The mean ACTH was 26.27 pg/mL in Treatment 2, which was 64.5% higher than that of Treatment 1 (15.97 pg/mL) during the summer. During the winter, ACTH of Treatment 2 (36.61 pg/mL) was 21% higher than that of Treatment 1 (30.24 pg/mL). Such results are consistent with the observed values of ADG, a direct productivity parameter. As a side note, significantly cold environments were observed to result in severe pig stress.
*Thermal and Gas Environment-Behavioral Response*: During summer, more pigs took a recumbent (P < 0.05) posture at moderate temperature ranges in Treatment 1, which is believed to result in higher ADG and FE. In hot, humid, and NH_3_-rich environment in Treatment 2, more pigs were lying (P < 0.01), due to severe environmental exhaustion. During winter, significantly more pigs were eating (P < 0.01), and lying in a lateral recumbent pose than at moderate temperature ranges. In Treatment 2, cold temperatures resulted in more pigs taking a sternal recumbent posture (P < 0.05), and few were standing (P < 0.05) or drinking (P < 0.05).

It is observed that the thermal and gas environments seriously influenced the productivities and degree of stress in terms of ADG, FE and ACTH for growing pigs in both seasons. In future studies, a representative indicator for pig welfare should be drawn and its relation with thermal and gas environments should be researched.

## Figures and Tables

**Table 1:** Comparison of environmental parameters between Treatments 1 and 2 during July 2003.

Parameters		Treatment 1			Treatment 2		t

		NS 1)	Mean	S.D. 2)	Mean	S.D.	
Thermal Environment	Air Speed (m/s)	10	2.25	0.07	0.06	0.005	96.98 ***
	Temp (°C)	10	26.45	0.018	32.23	0.15	−88.29 ***
	Humidity (%)	10	66.48	0.008	96.51	0.21	−270.48 ***

	AEI 3)	10	1758	81.9	2788	175.6	−204.3 ***

Gas Environment	NH_3_(ppm)	10	6.38	0.18	15.35	0.29	−80.37 **
	CO_2_ (ppm)	10	6528	10.04	11115	525.34	−27.61 ***

ACTH 4) (pg/mL)		10	15.97	5.55	26.27	7.01	−3.64 **

ADG 5) (g/day)		10	759.5	14.23	405.5	10.12	64.97 ***

FE 6) (D’less)		10	0.63	0.015	0.40	0.007	36.02 ***

Animal Behavior	Lying 7)	10	4.50	0.17	19.25	0.31	−129.11 ***
	Standing	10	9.28	0.13	2.36	0.33	61.24 ***
	Sitting	10	6.44	0.36	2.74	0.30	24.44 ***
	Drinking	10	10.19	0.24	11.63	0.49	−8.21 ***
	Eating	10	8.86	0.31	3.19	0.43	33.48 ***
	Fighting	10	0.68	0.41	0.80	0.28	−0.755

*** P < 0.001;

** P < 0.01;

* P < 0.05;

1) NS: Number of Samples;

2) SD: Standard Deviation;

3) AEI: Air Enthalpy Index;

4) ACTH: adrenocorticotropic hormone;

5) ADG: Average Daily Gain;

6) Feed Efficiency: weight gain/feed intake;

7) Lateral recumbency.

**Table 2:** Pearson correlation analysis of the environment, productivity, behavior parameters for Treatment 1 during summer.

Parameters	Temp.	R.H.	AEI	NH_3_	CO_2_	ACTH	ADG	FE	L	St	Si	D	E	F	
Thermal Environment	Temp.	1.00													
	R.H.	−0.148	1.00												

	AEI	0.964 **	0.108	1.00											

Gas Environment	NH_3_	−0.335	0.468	−0.217	1.00										
	CO_2_	0.226	0.065	0.224	0.205	1.00									

ACTH		0.225	0.010	0.224	−0.437	0.018	1.00								

ADG		0.149	−0.557	0.007	−0.074	0.686 *	0.035	1.00							
FE		0.643 *	0.489	0.607	0.558	0.810 **	0.079	0.739 *	1.00						

Behavior	Lying 1)	0.427	−0.622	0.270	−0.160	0.551	−0.316	0.702 *	0.707 *	1.00					
	Standing	0.173	−0.322	0.091	−0.189	0.143	0.374	0.507	0.364	0.255	1.00				
	Sitting	−0.669 *	0.001	−0.672 *	0.332	−0.346	−0.476	−0.071	−0.472	−0.188	0.190	1.00			
	Drinking	0.401	0.124	0.434	−0.188	−0.089	0.819 **	−0.186	0.098	−0.289	0.416	−0.320	1.00		
	Eating	0.791 **	0.267	0.864 **	−0.041	0.295	0.150	0.023	0.517	0.058	−0.100	−0.598	0.266	1.00	
	Fighting	−0.447	0.061	−0.434	0.000	−0.184	−0.216	−0.325	−0.457	−0.177	−0.747*	−0.173	−0.519	−0.372	1.00

*** p < 0.001;

** p < 0.01;

* p < 0.05;

1) lateral recumbency; L: refers to lying, St: standing, Si: sitting, D: drinking, E: eating, F: fighting.

**Table 3:** Pearson correlation analysis for the environments, production-related, behavior parameters for Treatment 2 during summer.

Parameters	Temp.	R.H.	AEI	NH_3_	CO_2_	ACTH	ADG	FE	L	St	Si	D	E	F	
Thermal Environment	Temp.	1.00													
	R.H.	−0.303	1.00												

	AEI	0.861 *	0.224	1.00											

Gas Environment	NH_3_	0.409	0.012	0.425	1.00										
	CO_2_	−0.349	0.257	−0.219	0.468	1.00									

ACTH		−0.645 *	0.087	−0.613	−0.319	−0.061	1.00								

ADG		0.274	−0.279	0.131	−0.294	−0.269	0.107	1.00							

FE		0.447	0.429	−0290	−0.580	0.068	0.253	0.258	1.00						

Behavior	Lying 1)	0.420	0.350	0.616	0.803**	0.207	−0.352	−0.424	−0.575	1.00					
	Standing	0.148	−0.009	0.146	−0.264	0.198	−0.242	0.699 *	0.617	−0.325	1.00				
	Sitting	−0.903**	0.018	−0.914**	−0.471	0.221	0.733 *	−0.228	0.440	−0.585	−0.214	1.00			
	Drinking	−0.314	0.198	−0.225	−0.151	−0.153	0.204	−0.444	0.231	−0.160	−0.330	0.344	1.00		
	Eating	0.522	−0.041	0.512	0.072	−0.267	−0.213	0.519	−0.366	0.113	0.138	−0.508	−0.737 *	1.00	
	Fighting	0.092	−0.702 *	−0.281	0.108	−0.034	−0.153	−0.122	−0.405	0.005	−0.215	−0.005	−0.427	0.012	1.00

*** p < 0.001;

** p < 0.01;

* p < 0.05;

1) lateral recumbency.

**Table 4:** Comparison of the environment parameters for Treatments 1 and 2, February 2004.

Parameters		Treatment 1			Treatment 2		t

		NS	Mean	S.D	Mean	S.D	
Thermal Environment	Air Speed (m/s)	8	0.178	0.46	0.89	0.24	−7.67 ***
	Temperature (°C)	8	19.52	0.46	10.66	1.10	20.96 ***
	Humidity (%)	8	75.3	11.0	51.90	14.0	3.71 ***
	AEI	8	1470	207	550	142	10.35 ***

Gas Environment	NH_3_ (ppm)	8	6.95	1.50	3.41	0.70	6.05 ***
	CO_2_ (ppm)	8	26.59	1113	920	399	4.16 ***

ACTH (pg/mL)		8	30.24	4.85	36.6	7.11	−2.09 ***

ADG (g/day)		8	862	22.4	799	23.4	5.42 ***

FE (D’less)		8	0.43	0.146	0.32	0.006	2.14 ***

Animal Behavior	Lying 1)	8	20.9	6.79	21.75	7.38	−0.247
	Standing	8	7.50	5.65	7.88	5.64	−0.133
	Sitting	8	5.25	1.38	4.125	2.23	1.210
	Drinking	8	3.00	1.51	2.63	1.06	0.574
	Eating	8	3.12	1.55	2.00	0.53	1.938
	Fighting	8	0.25	0.71	1.63	2.33	−1.60

*** p < 0.001;

** p < 0.01;

* p < 0.05;

1) sternal recumbency.

**Table 5:** Pearson correlation analysis of the environments, productivity, behavior parameters for Treatment 1 during winter.

Parameters	Temp.	R.H.	AEI	NH_3_	CO_2_	ACTH	ADG	FE	L	St	Si	D	E	F	
Thermal Environment	Temp.	1.00													
	R.H.	−0.335	1.00												

	AEI	−0.187	0.988 **	1.00											

Gas Environment	NH_3_	−0.585	0.578	0.500	1.00										
	CO_2_	−0.198	0.381	0.371	−0.283	1.00									

ACTH		−0.021	−0.491	−0.527	0.172	−0.433	1.00								
ADG		−0.068	−0.296	−0.303	−0.399	0.001	−0.301	1.00							

FE		0.286	0.239	0.300	−0.442	0.685	−0.650	−0.013	1.00						

Behavior	Lying 1)	0.042	−0.123	−0.118	0.023	−0.262	−0.073	0.040	−0.455	1.00					
	Standing	−0.141	0.476	0.471	0.165	0.322	−0.177	−0.050	0.478	−0.920 **	1.00				
	Sitting	−0.210	0.384	0.358	0.00	0.730 *	−0.039	−0.597	0.509	−0.435	0.436	1.00			
	Drinking	0.670	−0.631	−0.550	−0.644	−0.102	0.361	0.152	0.231	−0.487	0.200	−0.068	1.00		
	Eating	0.153	−0.895 **	−0.909 **	−0.323	−0.554	0.520	0.367	−0.317	−0.134	−0.187	−0.480	0.609	1.00	
	Fighting	−0.630	−0.061	−0.165	0.527	−0.058	0.280	0.056	−0.244	−0.052	−0.036	−0.073	−0.267	0.288	1.00

*** p < 0.001;

** p < 0.01;

* p < 0.05;

1) sternal recumbency.

**Table 6:** Pearson correlation analysis of the environments, productivity, behavior parameters in Treatment 2 during winter.

Parameters	Temp.	R.H.	AEI	NH_3_	CO_2_	ACTH	ADG	FE	L	St	Si	D	E	F	
Thermal Environment	Temp.	1.00													
	R.H.	−0.256	1.00												

	AEI	0.173	0.903 **	1.00											

Gas Environment	NH_3_	0.050	0.257	0.275	1.00										
	CO_2_	−0.551	0.763 *	0.852	0.321	1.00									

ACTH		0.165	−0.334	−0.277	0.599	−0.093	1.00								
ADG		0.741 *	−0.455	−0.130	0.159	−0.637	0.024	1.00							

FE		0.416	−0.509	−0.366	−0.526	−0.333	−0.178	0.406	1.00						

Behavior	Lying 1)	−0.175	−0.385	−0.445	0.146	−0.018	0.774 *	−0.347	−0.162	1.00					
	Standing	−0.414	0.479	0.291	0.176	0.323	−0.829 *	−0.083	−0.083	−0.820 *	1.00				
	Sitting	0.264	−0.085	−0.048	−0.401	−0.183	−0.329	0.096	0.554	−0.518	0.296	1.00			
	Drinking	0.769 *	0.211	0.589	0.199	−0.232	−0.093	0.590	−0.042	−0.324	−0.128	−0.158	1.00		
	Eating	0.582	0.000	0.285	−0.152	−0.535	−0.423	0.571	−0.059	−0.543	0.189	0.120	0.756	1.00	
	Fighting	0.824 *	0.045	0.419	0.291	−0.322	0.010	0.812	0.216	−0.414	−0.091	0.010	0.861 **	0.574	1.00

*** p < 0.001;

** p < 0.01;

* p < 0.05;

1) sternal recumbency.

## References

[1] Nienaber JA, Hahn GL, Yen JT (1987). Thermal environment effects on growing-finishing swine Part I—Growth, feed intake and heat production. Trans ASABE.

[2] Lemay SP, Guo H, Barber EM, Chenard L (2001). Performance and carcass quality of growing-finishing pigs submitted to reduce nocturnal temperature. Trans ASABE.

[3] Rafai P, Papp Z (1976). Influence of enthalpy and air humidity on heat equilibrium and heat production in fattening pigs. Acta Vet Acad Sci.

[4] Addis PB, Johnson HR, Heidenreich CJ, Jones HW, Judge MD (1967). Effect of humidity level in a warm growing environment on porcine carcass composition and quality. J Anim Sci.

[5] Christison GI (1988). Effect of fluctuating temperatures and humidity on growing pigs.

[6] Wathes CM, Jones JB, Kristensen HH, Jones EKM, Webster AJF (2002). Aversion of pigs and domestic fowl to atmospheric ammonia. Trans ASABE.

[7] Dennis MJ (1986). The effect of temperature and humidity on some animal disease—A review. Br Vet J.

[8] Morrision WD, Pirie PD, Perkins S, Braithwaite LA, Smith JH, Waterfall D, Doucett CM Gases and respirable dust in confinement buildings and the response of animals to such airborne contaminants.

[9] Donham KJ (1991). Association of environmental air contaminants with disease and productivity in swine. Am J Vet Res.

[10] Stombaugh DP, Teague HS, Roller WL (1969). Effects of atmospheric ammonia on the pig. J Anim Sci.

[11] Massabie P, Grainer R, Dividich SL (1997). Effects on environment conditions on the performance of growing-finishing pig.

[12] Massabie P, Grainer R Effect of air movement and ambient temperature on the zootechnical performance and behavior of growing-finishing pigs.

[13] Xin H, Shao B Real-time assessment of swiner thermal comfort by computer vision.

[14] Makoto A (1991). Windowless Livestock Structures.

